# Supplementary material for: Development of a computer-aided design software for the quantitative evaluation of aesthetic damage
Source: PLoS One. 2019 Dec 18;14(12):e0226322. doi: 10.1371/journal.pone.0226322 (PMC6919621; doi:10.1371/journal.pone.0226322)
Supplement: S1 Code site — 1. (ZIP) [file pone.0226322.s001.zip › [EESC_jr][Projeto]Relatorio_07_21/reports/templates_zip/backyard-high-converting-landing-page-template.zip!/backyard/documentation/documentation.html]

Backyard Documentation


## Documentation

Toggle navigation

- Color
- Navigation
- Logo
- Background
- Mailchimp
- Icon
- Contact
- Support

#### Start

Thank you for downloading Backyard Landing Page Template. Please use this help file to be your guide. If you have any questions, please drop us an email to mazagung@gmail.com.

#### Change Color

Check `<head>` part of the index.html. You will find all colors link available. Blue (default) color is active. So if you want to use other color, just remove the comment to make other color active.

#### Navigation

To change menu site or navigation, simply edit them on navigation section.

#### Change Logo

**1. Header Logo** 
Find "logo.png" in landing section, replace with your own image.

**2. Navigation Logo** 
Find "logo2.png" in navigation section, replace with your own image.

#### Change Background Image

Backyard Template use image as background in **header, download, and contact** section. To change background image, find "bg.jpg" and replace with your own image.

#### Mailchimp Email Subscription

Backyard Template use Mailchimp as default email subscription service. To activate your Mailchimp list, find signup form and edit action with your Mailchimp URL subscription. The first value beneath the form action URL is the identifier to link to your MailChimp account. The second value is your list's unique id. Both values are necessary to make sure your form submits to the proper list.
Next step is locate each of the form's field inputs and copy/paste the "name" for each input to the corresponding field.

#### Icon

We use beautiful icon sets by FontAwesome and Stroke 7 Icon Font by Pixeden. These icon font sets can be styled dynamically using CSS.
See full list of FontAwesome icons here: http://www.fontawesome.io/icons
Complete list of Stroke 7 Icon here http://themes-pixeden.com/font-demos/7-stroke/index.html

#### Contact

Backyard's contact form use working form. To activate your email, simply open **"contactengine.php"** and edit **"your@email.com"** with your own email. To make sure it's work, try to test send a message from front end.

#### Support

If you have any questions, please drop us an email to mazagung@gmail.com.

#### Credits

- Bootstrap
- Icon Set by FontAwesome
- Stroke 7 Icon by Pixeden
- Images by Unsplash and Pixabay
- App Screenshot by GraphicBurger
- Apple Devices Mockup by Pixeden
- Parallax Background by Minimit
- Owl Carousel by Owl Graphic
- Sticky Plugin by Anthony Garand
- Wow animation by Matt Aussaguel and Animate.css


---

Copyright Backyard 2015
